# Inhibition of Aurora-A improves insulin resistance by ameliorating islet inflammation and controlling interleukin-6 in a diabetic mouse model

**DOI:** 10.1080/21623945.2020.1829851

**Published:** 2020-10-11

**Authors:** Fandong Meng, Qiangwei Sun, Dongmei Zhou, Qiang Li, Jing Han, Deshan Liu, Jing Yang

**Affiliations:** a Department of Endocrinology, Affiliated Hospital of Xuzhou Medical University; b Department of Endocrinology, Wuhan Third Hospital, Wuhan, China; c Department of Plastic Surgery, Affiliated Hospital of Xuzhou Medical University; d Jiangsu Province Key Laboratory of Immunity and Metabolism, Department of Pathogenic Biology and Immunology, Xuzhou Medical University; e Department of Traditional Chinese Medicine, Qilu Hospital, Shandong University, Jinan, China

**Keywords:** Aurora-A, IL-6, macrophages, insulin resistance, inflammation

## Abstract

Aurora-A kinase, a serine/threonine mitotic kinase, is reportedly upregulated in skin tissues of individuals with type 2 diabetes mellitus , although its function in diabetes is unclear. C57BL/6 J mice were utilized to establish a type 2 diabetic model and explore the functions of Aurora-A in diabetes. Aurora-A was highly expressed in the pancreas of the diabetic mice as confirmed by western blot. Inhibition of Aurora-A did not affect fasting blood glucose and body weight, but did improve insulin resistance, as indicated by improved oral glucose tolerance, insulin tolerance, and the Homoeostasis Model Assessment-Insulin Resistance index. Blockade of Aurora-A dramatically decreased the number of infiltrating macrophages in the pancreas in parallel with decreases in the levels of serum insulin and interleukin-6 (IL-6) mRNA. The levels of phosphorylated forms of protein kinase B, which are the key mediators of in insulin resistance, were not induced in liver, adipocyte tissues, and skeletal muscle by alisertib treatment. Our findings indicate that suppression of Aurora-A could at least partially enhance insulin sensitivity by decreasing the number of infiltrating macrophages and IL-6 level in a type 2 diabetic mouse model.

## Introduction

Diabetes, which is characterized by hyperglycaemia, is one of the major metabolic disorders in the present century. Prolonged hyperglycaemia causes a series of fatal complications [1]. Global projections of diabetes in 2017 estimated that 700 million adults worldwide would have diabetes by 2045. There are already 425 million diabetic adults [2]. A combination of peripheral insulin resistance (IR) and dysfunctional insulin secretion by pancreatic β-cells is implicated in the pathogenesis of type 2 diabetes mellitus (T2DM) [3]. Many epidemiological studies have shown that chronic subclinical inflammation may be associated with the transformation of IR and T2DM 53 [4,5].

IR refers to a decrease in insulin sensitivity. To maintain normal operation of the body, more insulin needs to be released, which leads to hyperinsulinemia [6]. IR is closely associated with the occurrence and development of T2DM. Recent studies have documented the interference with the insulin signal transduction pathway after the insulin receptor intersects with the signal transduction of inflammatory factors, and due to the inflammatory factors produced by non-specific inflammation interfere. These are the main molecular mechanisms of IR caused by inflammation. Given the continuing increase in the global diabetic population and the enormous impact of diabetes on patient health and welfare, there is an urgent need to define factors and mechanisms that contribute to IR in diabetes and to identify new therapeutic targets. Aurora-A is one of the three proteins in the Aurora family. Aurora-A localizes to centrosomes and spindle poles, and has been implicated in centrosome maturation and spindle assembly [7]. Elevated levels of Aurora-A are generally detected in human cancers and are associated with poor prognosis [8,9]. Aurora-A preferentially induces β-cell DNA replication and promotes rat β-cell replication and the mitotic cell cycle [10]. Additionally, Aurora-A is also involved in controlling nuclear factor-kappa B-mediated inflammatory through the protein kinase B (AKT) pathway as well as IĸB stabilization [11]. Recently, it was shown that aberrant expression of Aurora-A is increased in the skin tissues of diabetic patients [12].

The foregoing findings indicate that Aurora-A may be a novel target in the treatment of IR. To explore this, we presently detected the expression of Aurora-A and clarified its role in a mouse model of T2DM.

## Materials

### Reagents

Streptozotocin (STZ) was purchased from VICMED (Jiangsu, China). Neutral insulin injection solution was purchased from Wanbang (Jiangsu, China).

### Equipments

Equipments used were detailed in the following subsections.

### Experimental animals

Four-week-old male C57BL/6 J mice obtained from Vital River (Beijing, China) were housed under environmentally controlled conditions. All experimental procedures complied with the Provision and General Recommendation of the Chinese Laboratory Association and were approved by the Institutional Animal Care and Use Committee of Xuzhou Medical University.

### Induction of diabetes in mice and drug administration

After adjusting to the laboratory environment for 7 days prior to the experiments, mice were fed normal chow (Normal) or a high-fat diet (HFD) for 7 days. The HFD contained 60% fat and 40% standard chow (Research Diets Inc., New Brunswick, NJ, USA). The mice were fasted for 16 h before receiving STZ. STZ was dissolved in freshly prepared 100 mM citrate buffer (pH 4.5) and administered by intraperitoneal injection at 85 mg/kg on days 0 and 1. The Normal and HFD groups of mice were injected with the same amount of citrate buffer. During the injection, mice in the HFD group injected with STZ were orally administered 5% glucose (2 mL/kg body weight) 24 h after each injection to prevent initial mortality due to STZ-induced hypoglycaemia.

Mice displaying fasting plasma glucose (FPG) levels higher than 11.1 mM [13] were considered HFD/STZ-diabetic mice. FPG levels and body weight were monitored once a week for 5 weeks. To assess the role of Aurora-A in the diabetic model (DM), beginning at week 4 the DM group was orally treated daily with 20 mg/kg of alisertib, a novel oral Aurora-A inhibitor, for 2 weeks. The N and HFD groups received the same amount of normal saline every day. During the trial, individual body weight and FBG levels were measured once a week. At the termination of the study, the mice were sacrificed. Blood samples were collected from the vein behind the eye sockets and stored at −80°C until analyses. Pancreatic samples were immediately acquired and stored at −80°C until required.

### FPG concentration

Blood (0.1 mL) was collected from the orbital vein and orbital artery of mice after 16 h of fasting and before their sacrifice. The PG concentration was measured 16 h after removal of food at 7 p.m. This represented the FPG concentration. The determination was made using a blood glucose device (Sanwa Kagaku, Nagoya, Japan). Immediately after the measurement was made, mice were provided with food.

### Oral glucose tolerance test (OGTT)

Mice were fasted for 16 h before oral administration of glucose solution (2.0 g/kg). PG concentration was measured 30 min prior to glucose administration (‘Pre’) and 15, 30, 60, and 120 min after glucose administration on day 35 after induction of diabetes.

### Insulin tolerance test (ITT)

Mice were fasted for 16 h before the subcutaneous injection of insulin (0.04 U/10 g of body weight). PG concentration was measured 30 min prior to insulin injection (‘Pre’) and 30, 60, and 120 min after the injection of insulin on day 36 after induction of diabetes. Insulin (Penfil R, 100 U/mL) was diluted with insulin dilution buffer (10 mM HCl solution containing 0.1–0.25% phenol and 1.4–1.8% glycerine) according to the manufacturer’s instructions.

### Measurement of insulin and other biochemical parameters

Insulin and other biochemical parameters were measured at the end of the study. Briefly, blood samples were collected from the ocular artery 16 h after fasting. The serum was used to detect the levels of insulin, total cholesterol (TC), and triglycerides (TG). Serum insulin levels were measured using a Mouse Insulin ELISA kit (BioVision Inc., Milpitas, CA, USA). TG and TC were measured with respective kits (Jiancheng, Nanjing, China). The Homoeostasis Model Assessment of Insulin Resistance (HOMA-IR) was calculated as (FPG [mmol/L] × fasting plasma insulin [uUI/ml])/22.5.

### Mouse islet isolation and Ki-67 staining

Islets were isolated from the pancreas of mice after collagen digestion as described previously [14]. Briefly, the pancreas was injected with 5  mL of cold enzyme solution (Hank’s Balanced Salt Solution containing 25 mM HEPES buffer and collagenase Type V; Sigma-Aldrich, St. Louis, MO, USA). The pancreas was removed from each mouse and digested at 37°C for 18 min. Enzymatic activity was terminated following the addition of cold (4°C) 10% RPMI. Islets were then isolated by centrifugation, decanting the supernatant, resuspension, and filtering through a 450-μm screen. Finally, islets were purified by Euro-Ficoll density gradient centrifugation. The islet cells were fixed in 4% paraformaldehyde, permeabilized in permeabilization buffer (0.02% Triton-100 in PBS), blocked in blocking solution (0.5% BSA in PBS), and then incubated with rabbit anti-Ki-67 (1:100) (D3B5, Cell Signalling Technology, #12,202) antibody. Fluorescein isothiocynate (FITC)-conjugated goat anti-rabbit IgG (1:200; VICMED) was used as the secondary antibody. Fluorescence was imaged using an LSM 700 laser scanning confocal fluorescent microscope (Carl Zeiss, Jena, Germany).

### Tissue preparation and western blot

Western blot was performed as described previously [15]. The frozen pancreatic tissues were cut into pieces and homogenized in ice-cold cellular lysis buffer (150 mM NaCl, 10 mM Tris–HCl, 5 mM EDTA, 1 mM EGTA, and 10% Triton X-100) containing a protease inhibitor cocktail. The homogenates were sonicated for 5 min. The tissue lysates were centrifuged for 10 min at 13,200 rpm at 4°C. The tissue protein concentrations were measured using the Bradford protein assay. Equal amounts of total protein from the tissue lysates were loaded and resolved by 10% sodium dodecyl sulphate-polyacrylamide gel electrophoresis (SDS-PAGE). The proteins were transferred to nitrocellulose membranes (Millipore, Billerica, MA, USA). After blocking in 5% non-fat milk for 30 min at room temperature, the membranes were incubated with the indicated antibodies at 4°C overnight. The membranes were incubated with the appropriate horseradish peroxidase (HRP)-conjugated secondary antibody for 2 h at room temperature. HRP activity was visualized using ClarityTM Western ECL Substrate and a ChemiDoc™ MP Imaging System (Bio-Rad Laboratories, Hercules, CA, USA). Densitometric analysis was performed using Image LabTM software version 5.1 (Bio-Rad Laboratories).

### Haematoxylin and eosin (H&E) and immunofluorescence staining

For H&E staining, the histological sections were deparaffinized and hydrated through a series of decreasing concentrations of ethanol (100–80%), then stained with H&E (Beyotime, Shanghai, China), and covered with synthetic resin. Samples were observed by light microscopy using an Eclipse E400 microscope (Nikon, Tokyo, Japan) as described previously [16].

Islet inflammation was assessed by quantifying the number of macrophages and neutrophils within the islets using a PANO 3-plex IHC kit (YX Biotechnology, Shanghai, China). Different primary antibodies were sequentially applied, followed by HRP-conjugated secondary antibody and tyramide signal amplification (TSA). Nuclei were stained with 4ʹ-6ʹ-diamidino-2-phenylindole (DAPI; Sigma-Aldrich, St. Louis, MO, USA). Quantification of the positively stained cells was verified from 15 to 20 islets/group. The mean number from all fields of each pancreatic sample was calculated.

### RNA extraction and quantitative RT-PCR (qPCR)

Total RNA was isolated from the islet tissue using TRIzol reagent according to the standard procedure (Invitrogen, Carlsbad, CA, USA), followed by real-time RT-PCR with One Step TB Green™ PrimeScript™ RT-PCR Kit II (TaKaRa Bio, Shiga, Japan). Primers designed with Primer Premier 5.0 were as follows (F denotes forward and R denotes reverse): interleukin-6 (IL-6)-F, 5ʹ-CCGGAGAGGAGACTTCACAG-3ʹ; IL-6-R, 5ʹ-TCCACGATTTCCCAGAGAAC-3ʹ; β-actin-F, 5′-GCTACAGCTTCACCACCACA-3; and β-actin-R, 5′-TCTCCAGGGAGGAAGAGGAT-3ʹ. Results were normalized to β-actin mRNA.

### Statistical analyses

Data were analysed using SPSS software (version 16.0; SPSS Inc., Cary, NC, USA). Comparisons of two groups are presented as mean ± SEM. The 2-tailed paired t-test was used for data that were simultaneously normally distributed and homogenously variant. The Mann-Whitney test was used for data that were not. A pairwise comparison between groups was conducted by one-way ANOVA with appropriate Bonferroni’s multiple comparison test. A *P*-value < 0.05 was considered statistically significant.

## Results

### Establishment of mouse model of T2DM by combined administration of STZ and HFD

**Figure 1.**
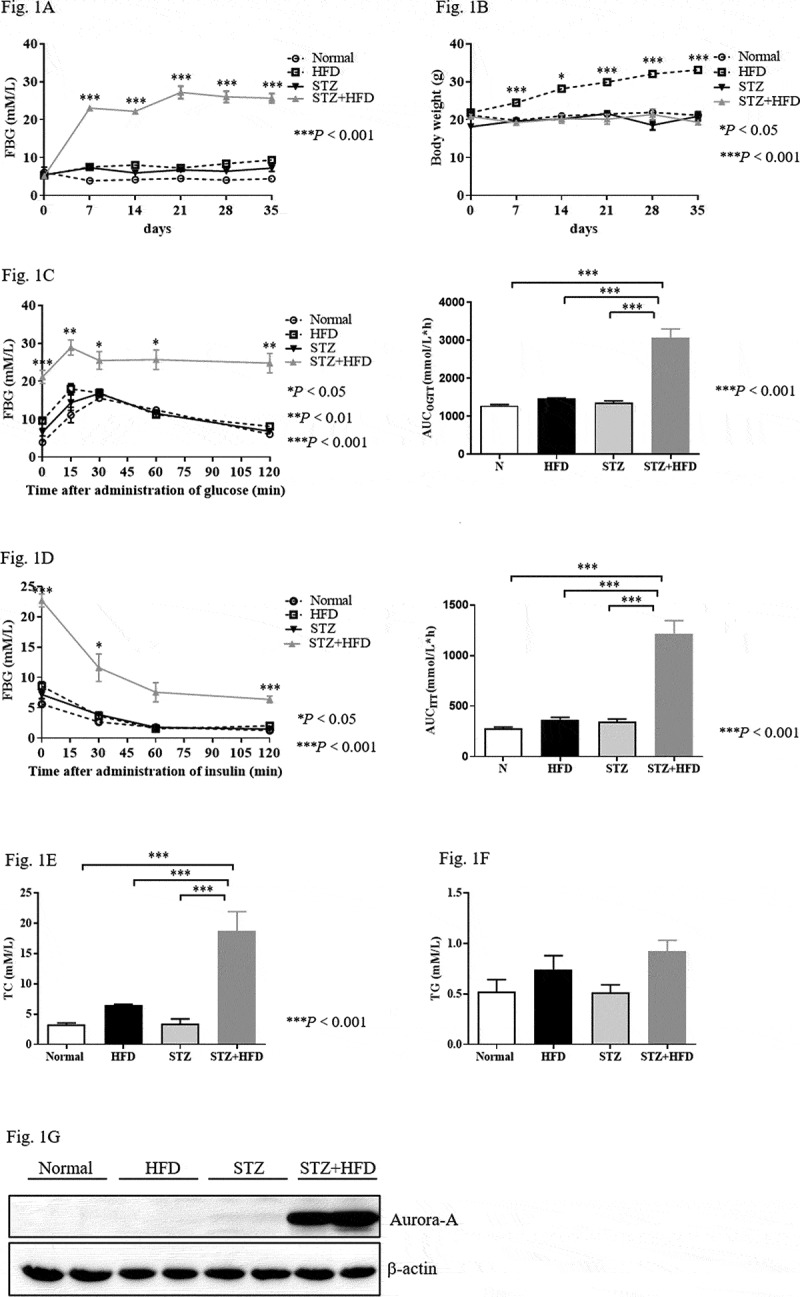
High expression of Aurora-A in the diabetic mouse model established with STZ and HFD

Seven days after the injection of STZ, the fasting blood glucose of mice in the STZ+HFD group was significantly higher than that in the normal, STZ alone, and HFD groups (Figure 1(a), *P* < 0.01). Conversely, without obvious changes in body weight in STZ+HFD mice, the body weight of the HFD group was increased compared with that of the normal group and STZ alone group (Figure 1(b)). During the experiment, loss of hair lustre, polydipsia, polyuria, and polyphagia were also observed in the diabetic mice (data not shown).

Male 5-week-old C57BL/6 J mice were fed normal chow or the HFD. The mice were randomly divided into four groups. The individual FPG concentration (a) and body weight (b) were measured once a week for 35 days after two injections of with STZ (85 mg/kg). Mice that consumed the normal diet were used as the control. Data are expressed as mean±S.E.M. Analysis involved one-way ANOVA with appropriate Bonferroni’s multiple comparison test. Significance is indicated as *P < 0.05, and ***P < 0.001 vs. the normal group. Blood glucose levels during the OGTT (c) or ITT (d) and the AUC of the blood glucose were measured at the end of the experiment after the induction of diabetes. Data are expressed as mean±S.E.M. Analysis involved one-way ANOVA with appropriate Bonferroni’s multiple comparison test. Significance is indicated as *P < 0.05, **P < 0.01, and ***P < 0.001 vs. the normal group. Serum from normal mice, HFD mice, or diabetic mice was collected before their sacrifice. Levels of TC (e) and TG (f) were examined. Data are expressed as mean ± S.E.M. Analysis involved one-way ANOVA with appropriate Bonferroni’s multiple comparison test. Significance is indicated as***P < 0.001 vs. the normal group. (g) Expression of Aurora-A in the indicated mice was examined by western blot.

Oral glucose tolerance was assessed using the OGTT. An increase in the area under curve (AUC) was observed in the STZ+HFD group compared to the normal, STZ alone, and HFD groups. However, no obvious difference was found between the normal, STZ, and HFD groups (Figure 1(c), right panel). In the ITT, the STZ+HFD group displayed decreased in FPG during the first 60 min after the injection of insulin (Figure 1(d)). Then, the FPG concentration was maintained at 5 to 7 mM. However, the levels of FPG were maintained at almost the original levels prior to the administration of insulin in the normal, STZ alone, and HFD groups (Figure 1(d), left panel). In addition, the AUC was significantly increased in the STZ+HFD group compared with the other three groups (Figure 1(d), right panel). Serum TC was significantly increased in the diabetic mice, although the levels of TG were identical in mice treated with STZ+HFD compared with mice treated solely with STZ or the HFD (Figure 1(e,f)). The findings indicated that the diabetic mice suffered from hypercholesterolemia.

### Overexpression of Aurora-A in pancreas of diabetic mice

High levels of Aurora-A expression have been observed in diabetic patients [17]. We examined the expression of Aurora-A in the aforementioned diabetic mouse model. Similar with prior data, the levels of Aurora-A were significantly increased in the pancreas of the STZ+HFD group compared with those of the normal, STZ, and HFD groups (Figure 1(g)).

### Aurora-A inhibition does not affect body weight or blood glucose in diabetic mice

**Figure 2.**
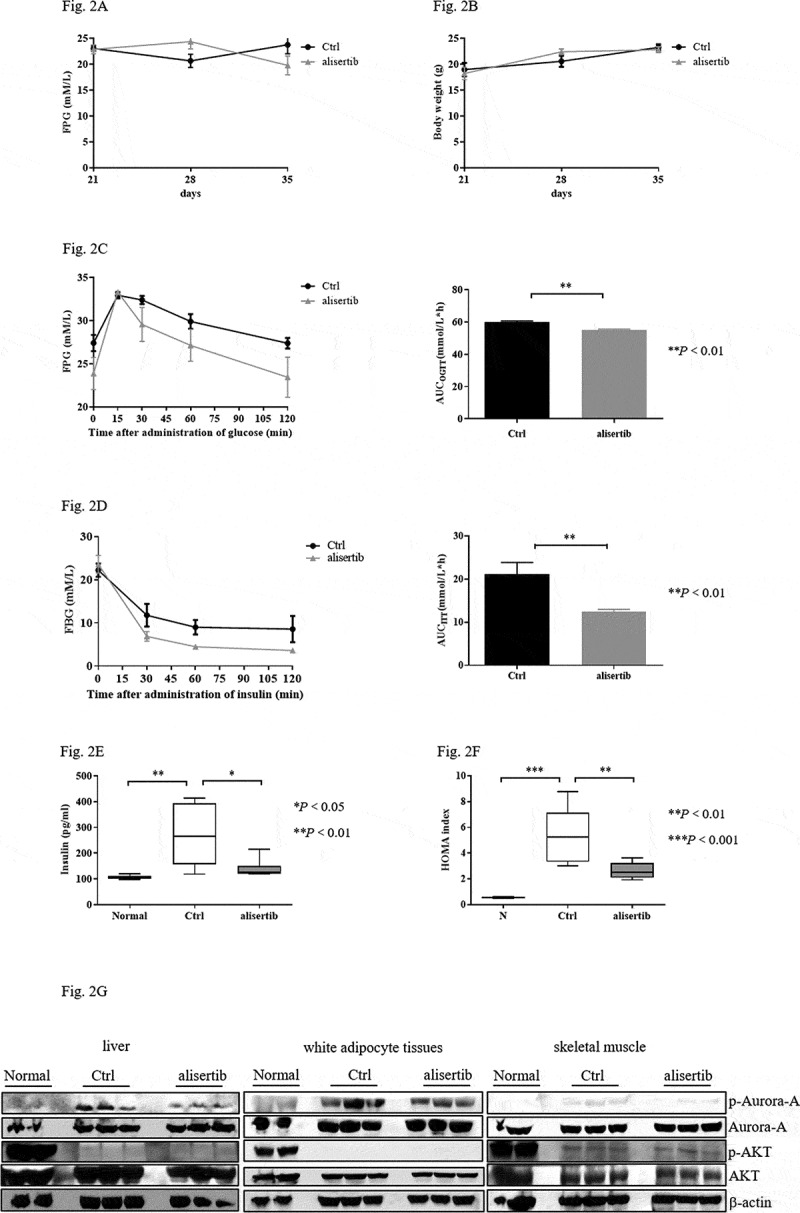
Suppression of Aurora-A improves insulin resistance (IR) in STZ+HFD mice

To elucidate the role of Aurora-A in diabetic mice, STZ+HFD mice were also treated with alisertib (20 mg/kg) or the same volume of saline (control group) for 2 weeks. FPG and body weight were monitored. FPG levels in the alisertib-treated diabetic mice were identical to those in control mice (Figure 2(a)). Body weight was not affected by alisertib compared with the control group (Figure 2(b)).

Fasting FPG (a) and body weight (b) during alisertib treatment. Diabetic mice treated with normal saline served as the control. Data are expressed as mean ± S.E.M and were analysed using the 2-tailed paired t-test. Blood levels measured by OGTT (c) or ITT (d) and the AUC of blood glucose were assessed after 2 weeks of treatment with alisertib. Data are expressed as mean±S.E.M and were analysed using the 2-tailed Mann-Whitney test. Significance is indicated as **P < 0.01, vs. control group. Serum levels of insulin (e) were determined. Data are expressed as mean ± S.E.M, one-way ANOVA with appropriate Bonferroni’s multiple comparison test, *P < 0.05, **P < 0.01, vs. control group, n＝6 per group. HOMA-IR index (f) of normal, control, and alisertib groups. Data are expressed as mean ± S.E.M. The analysis involved one-way ANOVA with appropriate Bonferroni’s multiple comparison test. Significance is expressed as **P < 0.01 and ***P < 0.001 vs. control group, n＝6 per group.

### Suppression of Aurora-A improves IR in diabetic mice

To further assess whether Aurora-A was involved in regulating IR by improving glucose tolerance and insulin tolerance in STZ+HFD mice, OGTT and ITT were performed after the 2-week alisertib treatment. In the OGTT, decreased AUC was observed in mice treated with alisertib after glucose administration (Figure 2(c)). Alisertib also significantly decreased blood glucose levels after insulin injection compared with that of the control group, as evident by the decreased AUC (Figure 2(d)). Serum insulin in these STZ+HFD mice with and without alisertib treatment revealed markedly high levels of insulin in the control group, with a profound reduction upon alisertib treatment (Figure 2(e)). The STZ+HFD mice showed a significant increase in the HOMA-IR index compared to the normal group. When these mice were treated with alisertib, the HOMA-IR index was significantly reduced (figure 2(f)).

Defects in the AKT/PKB pathway are associated with IR [18,19]. We investigated the levels of activated AKT. Alisertib treatment blocked Aurora-A activity. However, the levels of p-AKT were not increased in liver tissues compared to that in control mice (Figure 2(g)). Similarly, the levels of p-AKT were not affected in adipocyte tissues or skeletal muscle (Figure 2(g)).

### Blockade of Aurora-A may not affect β-cell proliferation in diabetic mice

**Figure 3.**
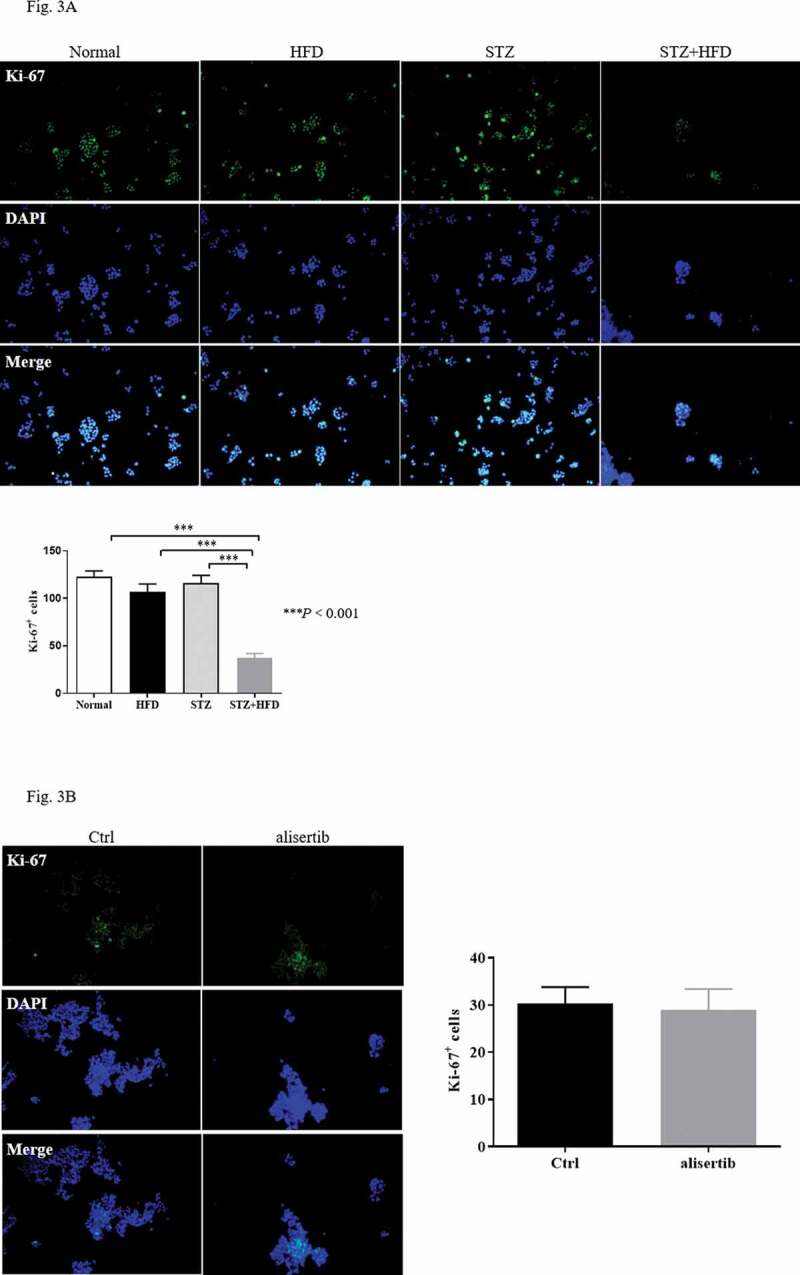
Blockade of Aurora-A does not affect proliferation of β-cells in diabetic mice

A previous study indicated that Aurora-A plays an important role in mediating β-cell proliferation [10]. We used Ki-67 staining to investigate the proliferation of pancreatic islet cells in diabetic mice. The number of Ki-67 positive islet cells in the STZ+HFD group was significantly decreased compared with that in the normal, STZ, and HFD groups (Figure 3(a)). Unexpectedly, alisertib treatment did not result in a change in the number of Ki-67+ islet cells in STZ+HFD mice compared with saline-treated SZT+HFD mice (Figure 3(b)). The finding indicated that the aberrant expression of Aurora-A might be important in controlling insulin sensitivity rather than mediating the proliferation of islet cells.

(a) Islet cells from normal, STZ, HFD or STZ+HFD mice were fixed, permeabilized, and stained with anti-Ki-67 antibody. Quantification of the Ki-67+ cells was verified from 10 to 12 random fields at 100× magnification. Data are expressed as mean ± S.E.M. The analysis involved the 2-tailed t-test. Significance is expressed as ***P < 0.01 vs. the normal group. (b) Islet cells from either control or alisertib-treated STZ+HFD mice were fixed, permeabilized, and stained with anti-Ki-67 antibody. Quantification of the Ki-67+ cells was verified from 10 to 12 random fields at 100× magnification. Data are expressed as mean ± S.E.M using the 2-tailed, t-test.

### Inhibition of Aurora-A suppresses macrophage infiltration

**Figure 4.**
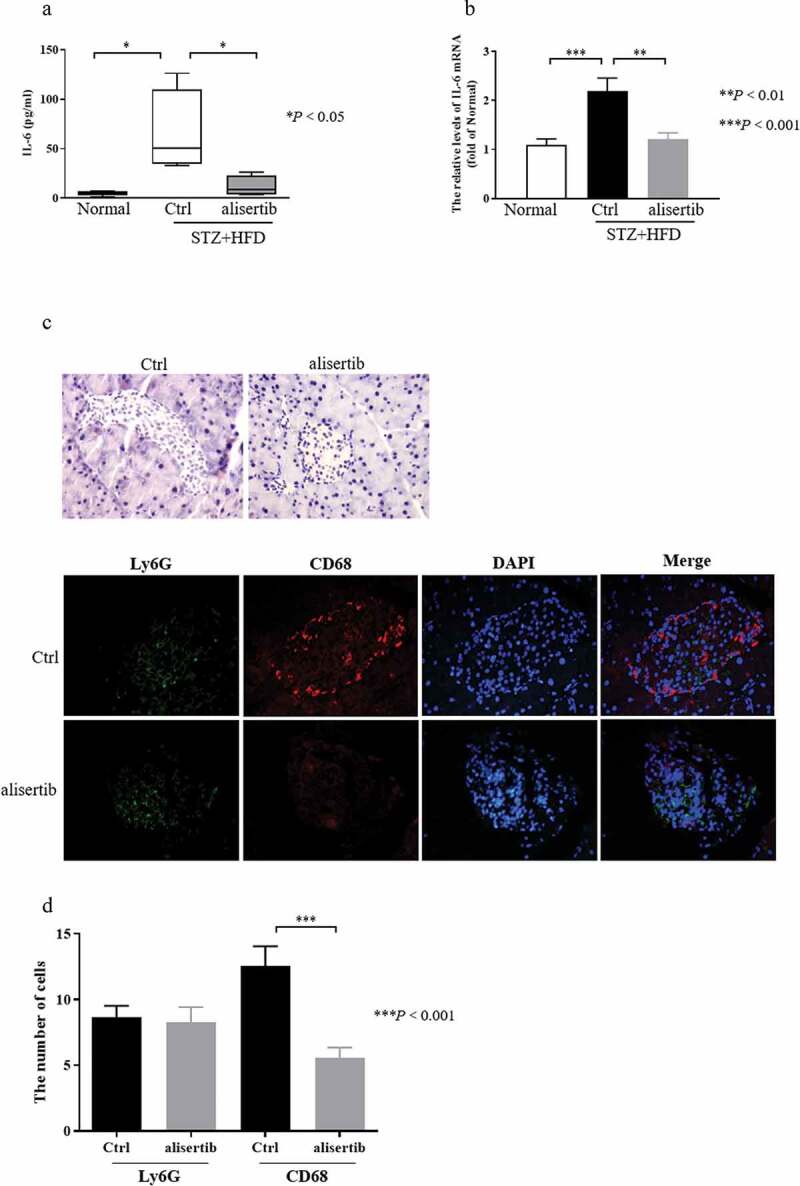
Alisertib inhibits macrophages infiltration and downregulates the levels of IL-6

Multiple studies have indicated that the levels of IL-6 are associated with hyperglycaemia and IR [17,20–23]. Therefore, to determine the mechanisms by which Aurora-A mediated insulin sensitivity, we examined levels IL-6 in serum and the expression of IL-6 in pancreatic tissue. As shown in Figure 4(a), the serum level of IL-6 in STZ+HFD mice was significantly higher than that in normal mice, whereas, in the presence of alisertib, the serum level of IL-6 in these mice was significantly lower. Consistent with the above results, the qPCR results showed that IL-6 mRNA levels were significantly higher in the STZ+HFD mice than in the normal mice (Figure 4(b)).

(a) The serum levels of IL-6 were examined by ELISA. Diabetic mice were treated with normal saline as control group. Data are expressed as mean ± S.E.M using one-way ANOVA with appropriate Bonferroni’s multiple comparison test. Significance is expressed as *P < 0.05 vs control group (n = 4–5). (b) The relative levels of IL-6 mRNA. The relative levels of IL-6 mRNA were detected by qPCR and normalized to β-actin in pancreatic tissue. Data are expressed as mean± S.E.M and analysed by one-way ANOVA with appropriate Bonferroni’s multiple comparison test. Significance is expressed as **P < 0.01 and ***P < 0.001 vs. the control group (n＝10-11). (c) Immunofluorescence by CD68 (red), Ly6G (green), and DAPI (blue) in pancreatic sections that were untreated or treated with alisertib. (d) Quantitation of CD68+ macrophages and Ly6G+ neutrophils in the islet sections that were untreated or treated with alisertib. Data are representative of one of two independent experiments with 15 to 20 islets/group.

However, the expression of IL-6 mRNA in STZ+HFD mice was obviously decreased following alisertib treatment (Figure 4(b)). IL-6 is predominantly produced by macrophages [24]. Additionally, macrophage infiltration is critical in mediating pancreatic islet inflammation during the progression of T2DM [25]. Based on this information, we quantified the number of macrophages and neutrophils within islets to assess the inflammatory status of islets. The number of infiltrating macrophages (CD68+ cells), but not neutrophils (Ly6G+ cells), were dramatically reduced in pancreatic tissues from alisertib-treated mice compared with the numbers in control mice (Figure 4(c,d)). 277

## Discussion

T2DM is a slowly progressive disease. An important cause of its occurrence and development is the decompensation of IR [26]. Improving IR is recognized as a potentially important treatment of T2DM. Aurora-A protein expression is elevated in diabetic skin tissue [12]. This discovery has prompted extensive research on the physiological significance of Aurora-A. Most of the studies have focussed on its relationship with cancer. Presently, the expression of Aurora-A was higher in the pancreatic tissue of the diabetic mice (Figure 1). A previous study demonstrated the involvement of Aurora-A in controlling the proliferation of β cell [10]. However, we observed that blocking Aurora-A activity using alisertib did not change the proliferation of islet cells (Figure 3(b)). We cannot rule out the possibility that our experiment missed the time point for islet β cell

proliferation after administration of STZ. Interestingly, we observed that the AUC of ITT and HOMA-IR index was significantly decreased in alisertib-treated STZ+HFD mice compared with the values in saline-treated STZ+HFD mice. Furthermore, the levels of serum insulin in STZ+HFD mice were significantly lower when they also were treated with alisertib compared to mice treated with saline. The findings indicated that the inhibition of Aurora-A might be involved in attenuating IR. The common ectopic fat deposits in obese patients are liver and skeletal muscle [27]. These sites are also common sites of IR [28]. Unfortunately, alisertib did not contribute to an increase in the levels of p-AKT in the liver and skeletal muscle (Figure 2(g)).These observations indicate that alisertib ameliorated IR by other mechanisms than AKT activation. We also found that the inhibition of Aurora-A did not affect the levels of FPG and the body weight of the diabetic mice. The data indicate the indirect involvement of Aurora-A in the inhibition of diabetes.

IR is a common metabolic state that may cause atherogenic dyslipidemia, obesity and T2DM. IR is also important in the pathogenesis of T2DM [29,30]. Activated inflammatory signals directly affect IR and trigger systemic IR [31,32]. IL-6, IL-1β, and tumour necrosis factor-alpha are proinflammatory cytokines with cytotoxic, cytostatic (inhibition of insulin synthesis and secretion), or cytocidal activities on pancreatic islets. These activities can promote the development of IR [33,34]. An investigation of T2DM has revealed the important role of IL-6 [35]. Circulating IL-6 levels are increased in insulin-resistant states, such as impaired glucose tolerance [36] and T2DM [37,38]. The circulating IL-6 levels correlate with the risk of developing T2DM. It was recently shown that IL-6 can induce IR and that IL-6 gene expression is markedly upregulated in the affected individuals [35]. Presently, the serum and pancreatic tissue levels of IL-6 were increased in STZ+HFD mice. IL-6 was significantly decreased after the administration of alisertib (Figure 4(a)). We speculate that the inhibition of Aurora-A could reduce IR by inhibiting the release of IL-6. IL-6 is predominantly produced by macrophages [24]. Of note, infiltrating macrophages can lead to pancreatic islet inflammation during the progression of T2DM [25]. We assessed the ability of alisertib to decrease macrophage infiltration. As expected, the treatment decreased the number of infiltrating macrophages in pancreatic tissues compared with that in control mice (Figure 4(c,d)).

In conclusion, the inhibition of Aurora-A improved IR in mice following the induction of diabetes by STZ+HFD. The mice displayed fewer infiltrating macrophages and IL-6 was at least partially downregulated.
